# Influence of uncorrected refractive error and unmet refractive error on visual impairment in a Brazilian population

**DOI:** 10.1186/1471-2415-14-84

**Published:** 2014-06-25

**Authors:** Fabio H Ferraz, José E Corrente, Paula Opromolla, Silvana A Schellini

**Affiliations:** 1 Ophthalmology Department, Faculdade de Medicina de Botucatu, Universidade Estadual Paulista - UNESP, Cep: 18618-970 Botucatu, SP, Brazil; 2 Biostatistics Department, Instituto de Biociências de Botucatu, Universidade Estadual Paulista - UNESP, Botucatu, Brazil

**Keywords:** Blindness, Visual impairment, Spectacles, Refractive errors, URE, UREN

## Abstract

**Background:**

The World Health Organization (WHO) definitions of blindness and visual impairment are widely based on best-corrected visual acuity excluding uncorrected refractive errors (URE) as a visual impairment cause. Recently, URE was included as a cause of visual impairment, thus emphasizing the burden of visual impairment due to refractive error (RE) worldwide is substantially higher. The purpose of the present study is to determine the reversal of visual impairment and blindness in the population correcting RE and possible associations between RE and individual characteristics.

**Methods:**

A cross-sectional study was conducted in nine counties of the western region of state of São Paulo, using systematic and random sampling of households between March 2004 and July 2005. Individuals aged more than 1 year old were included and were evaluated for demographic data, eye complaints, history, and eye exam, including no corrected visual acuity (NCVA), best corrected vision acuity (BCVA), automatic and manual refractive examination. The definition adopted for URE was applied to individuals with NCVA > 0.15 logMAR and BCVA ≤ 0.15 logMAR after refractive correction and unmet refractive error (UREN), individuals who had visual impairment or blindness (NCVA > 0.5 logMAR) and BCVA ≤ 0.5 logMAR after optical correction.

**Results:**

A total of 70.2% of subjects had normal NCVA. URE was detected in 13.8%. Prevalence of 4.6% of optically reversible low vision and 1.8% of blindness reversible by optical correction were found. UREN was detected in 6.5% of individuals, more frequently observed in women over the age of 50 and in higher RE carriers. Visual impairment related to eye diseases is not reversible with spectacles. Using multivariate analysis, associations between URE and UREN with regard to sex, age and RE was observed.

**Conclusion:**

RE is an important cause of reversible blindness and low vision in the Brazilian population.

## Background

Refractive error (RE) is a remediable cause of visual impairment (VI), which is considered to be a social burden with a simple and cost‒effective treatment. RE has been included as one of the five priorities of the World Health Organization (WHO) in the global initiative for eliminating avoidable blindness [1]. RE has severe social and economic effects on individuals and communities, restricting educational and employment opportunities of otherwise healthy individuals [2].

The WHO definitions of blindness and VI are widely based on best-corrected visual acuity (BCVA) excluding uncorrected RE (URE) as a VI cause. Recently, URE was included as a cause of VI, thus emphasizing the burden of VI due to RE worldwide is substantially higher. URE was considered to be responsible for VI in approximately 259 million persons, of whom approximately 42 million are considered blind with visual acuity less than 3/60 in the better eye [3].

Several population‒based surveys have reported URE or presenting corrected (with habitual correction) VA point out the enormous burden of URE as reversible and amenable to rehabilitation VI cause [4,5].

The prevalence of RE determined in a population study was 40%, and 25-40% of these subjects utilized optical correction; however, approximately 80% of these corrections were outdated [6].

Visual acuity (VA) with proper correction increased by at least one line in 54% of people who participated in the Baltimore Eye Study, and 7.5% experienced an increase of three lines or more [7].

As part of the National Health and Nutrition Examination Survey (NHANES), an evaluation of the USA population demonstrated that 83.3% of those with VI could achieve good VA (VA ≥ 20/40 in the better-seeing eye) with refractive correction [8].

A study of RE in Latinos from Arizona demonstrated that RE are associated with increasing age and female gender and that RE have been associated with decreased quality of life and limitations in vision-dependent activities, as shown by fewer opportunities for education and employment and reduced productivity, resulting in indices of marginalization [9].

According to other studies, more than 33% of those who need spectacles did not have an appropriate prescription, and more than 25% of these individuals could experience visual improvement with proper correction of their RE. Using appropriate spectacles improved VA by at least one line in 26.7% of the studied population and by as much as four lines or more in 5.9% (95% CI: 5.2% - 6.7%). The prevalence of URE was 7.1%; unmet need (UREN) was more likely to be observed among the elderly, less educated, and those with myopia, although the reasons behind the high rate of URE were not identified [10].

The spectacle coverage rate in rural and urban populations 30 years of age in Bangladesh was 25.2% [2]. This rate is lower than other reported values [10], although it is difficult to compare results because the sample population in other reports was urban and had a wider age range.

Although the definitions vary, there are primarily two definitions used in studies involving RE: “Met need” describes the subjects who had VA less than 6/12 in the better eye without correction but who achieved 6/12 or greater in the better eye with their current distance spectacles, and “unmet need” (UREN) represents the subjects who had VA less than 6/12 in the better eye without correction and could achieve 6/12 or greater in the better eye with correction but did not wear spectacles or did not achieve such correction with their current spectacles [2,11]. UREN includes persons in whom the optical correction is sufficient for removing the individuals from the category of VI.

The overall prevalence of RE in Latino adults living in Arizona was 64% in at least one eye and 51% in both eyes. Of subjects with RE in at least one eye, 35% had URE and 33% of those with RE could benefit from a new pair of spectacles [9].

A substantial proportion (34%) of the population from Tehran lacked proper spectacles for correcting RE, although a considerable percentage would greatly benefit from spectacles. In addition, UREN was more likely among the elderly, less educated, and those with myopia, although the reasons for the high rate of UREN were not identified [10].

The primary causes of low vision and blindness in a Brazilian city were URE, cataract, and retinal diseases. The primary cause of presenting low vision was RE (72.3%), and cataract was the most prevalent cause of blindness (50%). Low vision was observed in 5.2% (95% CI: 4.3–6.1) of the population, whereas blindness was observed in 2.2% (95% CI: 1.6–2.8) of the population. Unilateral presenting low vision and unilateral presenting blindness were found in 8.3% (95% CI: 7.2–9.5) and 3.7% (95% CI: 2.9–4.4) of the population, respectively. Best corrected low vision was noted in 1.3% of the population (95% CI: 0.9–1.7), and best corrected blindness was found in 0.4% of people (95% CI: 0.2–0.7) [12].

Blindness in another Brazilian region was approximately 0.5% (regional variations range from 0.25% to 0.75%) [13], and causes of VI were RE (42.7%), cataract (23.6%), age-related macular degeneration (5.4%) and glaucoma (4.0%) [14].

Population studies involving RE in Brazilians are scarce, and the impact of RE on VI has not been established. The purpose of this study was to determine the prevalence of low vision (VI and blindness) attributable to RE and the improvement of VA as a result of appropriate optical correction in a Brazilian population.

## Methods

### Sampling procedure

A population-based cross-sectional ophthalmic survey of households was conducted in the west region of state of Sao Paulo, Brazil. The eligible population consisted of permanent, non-institutionalized residents aged ≥ 1 to 90 years between March 2004 and July 2005. The current investigation followed the tenets of the Declaration of Helsinki and was approved by Ethical Committee of Faculdade de Medicina de Botucatu, state of Sao Paulo, Brazil. All of the participants provided written informed consent before participating in the study.

The region has sixty-eight municipalities, of which nine were included in the study. Table 1 shows the locations of the municipalities and the total populations in 2003.

**Table 1:** Spatial location, the estimated population for the year 2000 and sample size of each participating municipality

**Municipality**	Location*		Population**	**Sample (N)**
	**South latitude**	**West latitude**		
**Arandu**	23°08′11″	49°03′16″	6065	746
**Areiópolis**	22°40′09″	48°39′47″	10296	758
**Bofete**	23°05′53″	48°15′31″	7356	692
**Conchas**	23°00′48″	48°00′22″	14904	1013
**Itaí**	23°24′49″	49°05′34″	21039	1020
**Pereiras**	23°04′24″	47°58′32″	6226	895
**Pratânia**	22°48′34″	48°39′57″	3950	697
**Manduri**	23°00′10″	49°19′28″	8271	1020
**Taguaí**	23°27′07″	49°24′38″	7468	813

*Google Earth, 2012; **IBGE (Demographic census in the year 2000).

The sample initially consisted of 3,600 residences, and 3,012 were evaluated, which corresponded to 83.6% of the total sample.

The participants were selected using a random, stratified, household cluster sampling technique. The households to be evaluated were selected according to the local census data (Instituto Brasileiro de Geografia e Estatística, 2000): the first house was selected randomly; the next house was the sixth house on the even-numbered side of the street and so on. The randomly selected household received a letter of invitation to participate in the study. The individuals who agreed to participate were contacted by telephone to schedule an appointment. All occupants of the household were eligible to participate in the study. If there was no answer when the examiners contacted the household or if people refused to participate in the research, the first house to the right was selected. If the next household refused to participate, the first house to the left of the initial house was selected and so on.

### Data collection

A single survey team conducted the study and all of the data collection were conducted using a Mobile Ophthalmic Unit. All study personnel underwent training and all procedures were standardized before beginning the study. Specific observations were performed by 1–2 members of the team to minimize interobserver variability. Trained health care workers filled out a detailed questionnaire regarding the demographic data (sex, skin color, and age), wearing and availability of spectacles, family history of eye diseases and presence of eye abnormalities. Each participant received an ophthalmic examination in which VA was measured for the right eye followed by the left eye with a consistently illuminated Snellen chart with tumbling E within a light box placed 5 meters from the participant. The VA was retested with the patient’s existing refraction. If the corrected VA was less than 20/20, an objective cycloplegic refraction (using the dilatation protocols listed below) was performed and the BCVA was recorded using the refraction result. If the subject was unable to read the largest letter at 5 m with objective refraction, testing was repeated at 1 m. If the subject was unable to read the largest letter at 1 m, the VA was recorded as count fingers (CF), hand movements (HM), light perception (LP) or no light perception (NLP). Using statistical analysis, the VA was converted to the logarithm of the minimum angle of resolution (logMAR).

Slit lamp biomicroscopy (Shinn-Nippon, Japan) was performed, followed by one drop of 1% cyclopentolate in each eye once (for people aged 1–39 years) and refractive examination after 30 minutes or 1% tropicamide (in those aged ≥40 years) and fundus examination at the slit lamp utilizing a 90D Volk lens (Mentor, USA). Intraocular pressure was evaluated using a non-contact tonometer (Canon TX-F, Japan) and if the intraocular pressure was higher than 21 mmHg, the measurement was repeated using a Goldman tonometer (Haag-Streit, USA).

Autorefraction (Topcon KR-7000, Japan) was performed for all subjects independent of VA. A subjective refraction examination was performed by ophthalmologists using a phoropter (Topcon VT10, Japan), assisted by autorefraction data, and confirmed using a retinoscope (Welch Allyn, USA) for those persons with a VA below 20/20.

### Definitions of RE

The spherical equivalent (SE) was calculated as the spherical error plus half the cylindrical error. We adopted the definitions of RE from the Baltimore Eye Study [7] as follows: myopia is defined as SE ≤ −0.5 D, high myopia as ≤ −3.0D, hyperopia as SE ≥ +0.5 D, high hyperopia as ≥ +3.0 D and astigmatism as DC ≤ −0.5 D. Anisometropia is defined as a difference in SE between the right and left eyes of ≥1.0 D.

The WHO categories of vision loss were used to define blindness and severe visual impairment [1] and separated VA into the following four strata: VA ≥ 0.15 logMAR (0.7 Snellen) was considered to be normal VA; 0.15 logMAR (0.7 Snellen) < VA ≤ 0.5 logMAR (0.3 Snellen) was considered to be moderate vision impairment; 0.5 logMAR (0.3 Snellen) < VA ≤ 1.3 logMAR (0.05 Snellen) was considered to be severe visual impairment; and VA > 1.3 logMAR (0.05 Snellen) was considered to be blindness.

The definitions for characterizing the improvement of VA by refraction examination were as follows:

1) URE (uncorrected RE) was defined as subjects who presented VA > 0.15 logMAR (0.7 Snellen) in the better eye but achieved ≤0.15 logMAR BCVA after refractive correction in the better eye;

2) UREN (unmet need RE) was defined as subjects who had an uncorrected VA worse than 0.5 logMAR or 0.3 Snellen in the better eye and could achieve BCVA ≤ 0.5 logMAR after refractive correction of the better eye but did not wear spectacles or did not achieve such correction with their present spectacles [2].

The treatments considered for RE were expectant, such as optical correction with improvement of VA, VI despite adequate optical correction, and blindness even with spectacles.

### Statistical analysis

There was a high correlation between the right and left eye RE data (Spearman r = 0.88). The analysis of the right eye RE and their demographic associations produced similar results to those in whom left eye RE was associated with demographic variables; hence, the results relating to the better eye are reported. The statistical software package used was SPSS version 15.0 (SPSS for Windows Inc., Chicago, IL, USA).

A descriptive analysis was performed using the mean, median and respective measures of dispersion (standard deviation and interquartile range). The proportion and prevalence data are presented in graphs, adopting 95% Confidence Intervals (CIs) and p values (significant at the p < 0.05 level).

Univariate analysis was performed to determine the presence of an association between the variables, and multiple logistic regression analysis was used to fit the best model for independent variables (all of the key variables analyzed in univariate analysis were included in multivariate models) to determine the predictive factors for VI, correctable VI and UREN. Odds ratios (ORs) (presented with 95% CIs) were used in the univariate analysis of VI, correctable VI and UREN with key variables.

## Results

**Figure 1:**
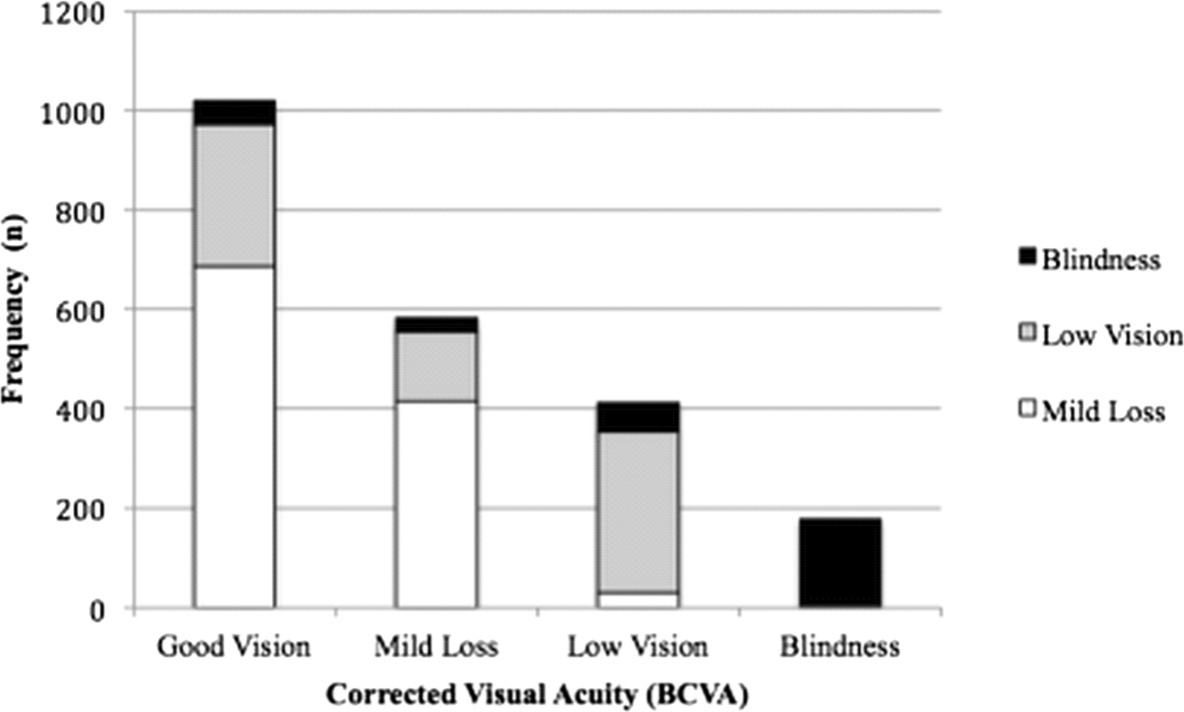
Distribution of uncorrected visual acuity categories (UCVA) according increased vision by spectacles (BCVA) in west region of state of São Paulo, Brazil – 2004/2005.

A total of 3,012 households were included in the study. A total of 8,010 subjects were selected, and 7,654 underwent ophthalmic examinations. The primary reasons for non-participation were work commitments, not meeting the inclusion criteria, and refusal to participate. Those who did not meet the inclusion criteria were women (62%), 3% below 40 years of age, 9% between 40 and 59 and 86% above. The municipalities were homogeneous for frequency of participation, ranging from 9.0% to 13.3%. The VA was measured in 7,362 individuals, and 70.2% presented with normal vision (UCVA ≤ 0.15logMAR), 15.4% presented with moderate VI (0.15logMAR < UCVA ≤ 0.5logMAR), 10.2% presented with severe VI (0.5logMAR > UCVA ≥ 1.3logMAR), and 4.2% presented with blindness (UCVA > 1.3logMAR).After best optical correction (BCVA), the frequency of individuals with normal VA increased to 84.1%, with a decrease in the frequencies of the other categories. This difference of 13.8% represents the prevalence of individuals with URE. With the correction of RE, 60.7% of individuals with moderate VI reached normal VA, and 15.7% of persons with severe VI reached the category of moderate loss. Approximately 18.9% of individuals who were considered to be blind with URE reached the level of low vision after optical correction (Figure 1).

**Figure 2:**
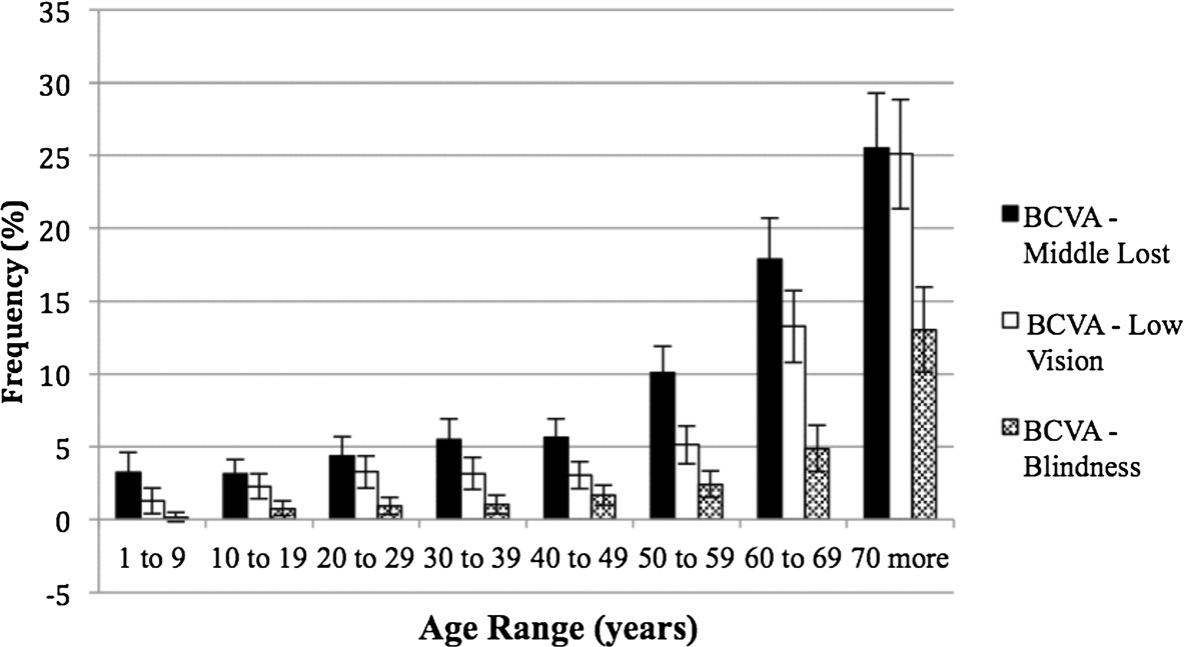
Frequency of best corrected vision acuity and no corrected vision acuity and its difference (URE), according to age in west region of state of São Paulo, Brazil – 2004/2005.

**Figure 3:**
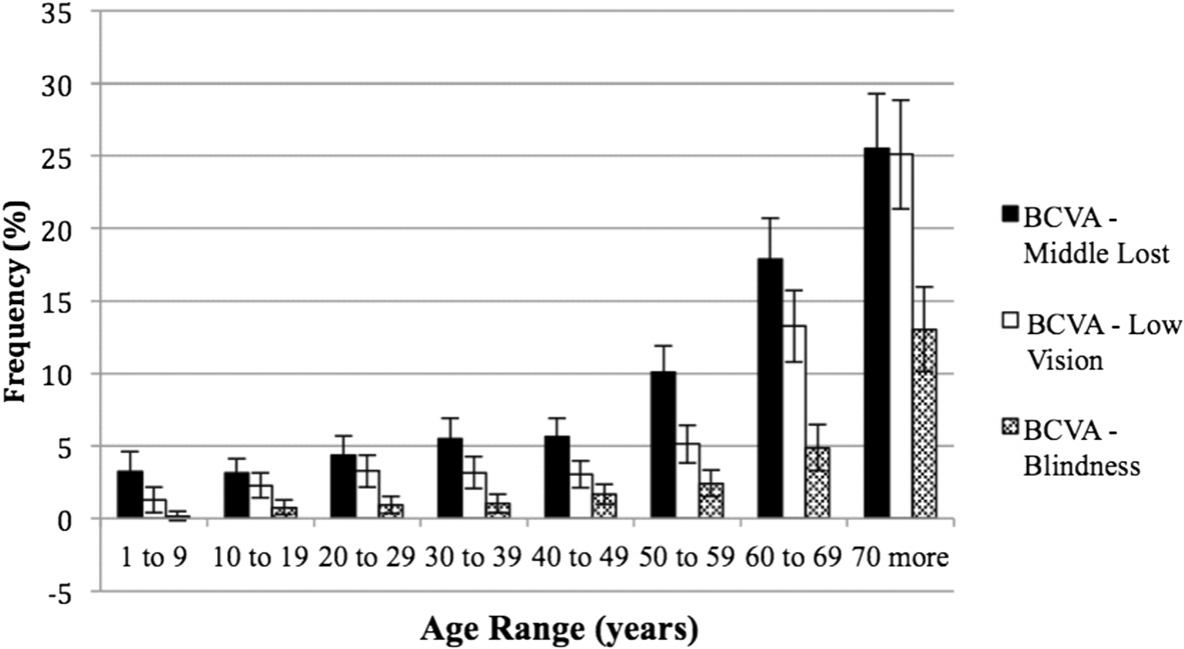
Relative frequency of visual acuity levels less than 0,15 logMAR after spectacles, according to age in west region of state of São Paulo, Brazil – 2004/2005.

**Figure 4:**
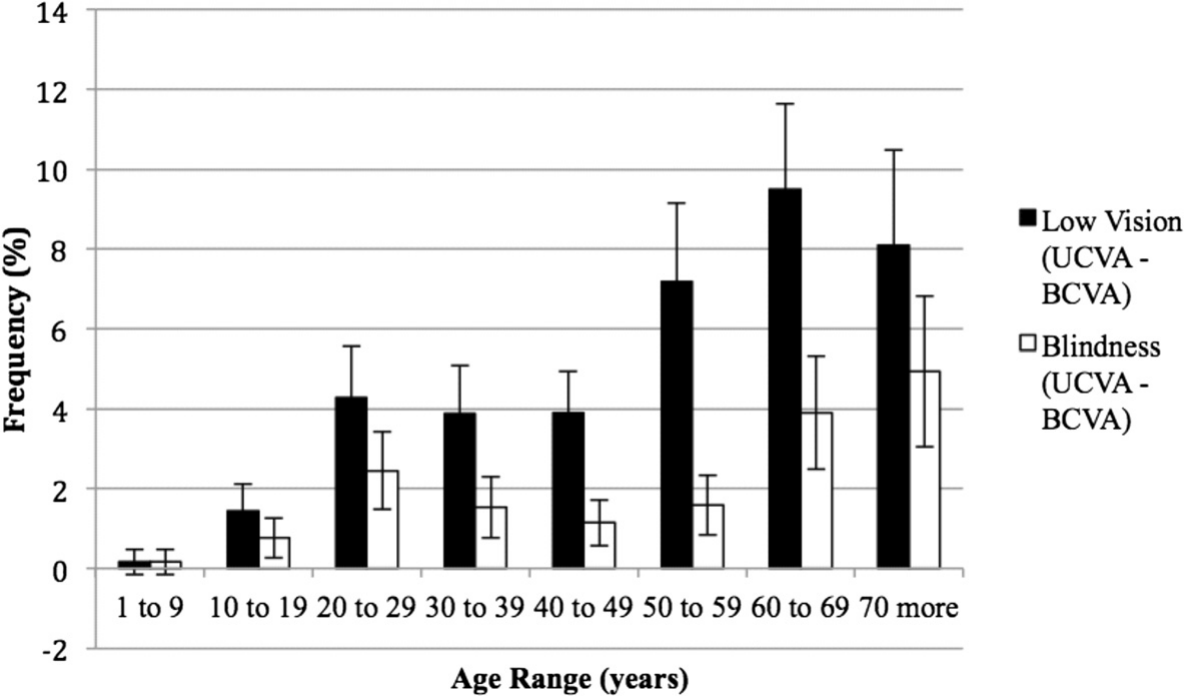
Prevalence and Confidence Interval (95%) of spectacle reversible visual impairment, according to age in west region of state of São Paulo, Brazil – 2004/2005.

**Figure 5:**
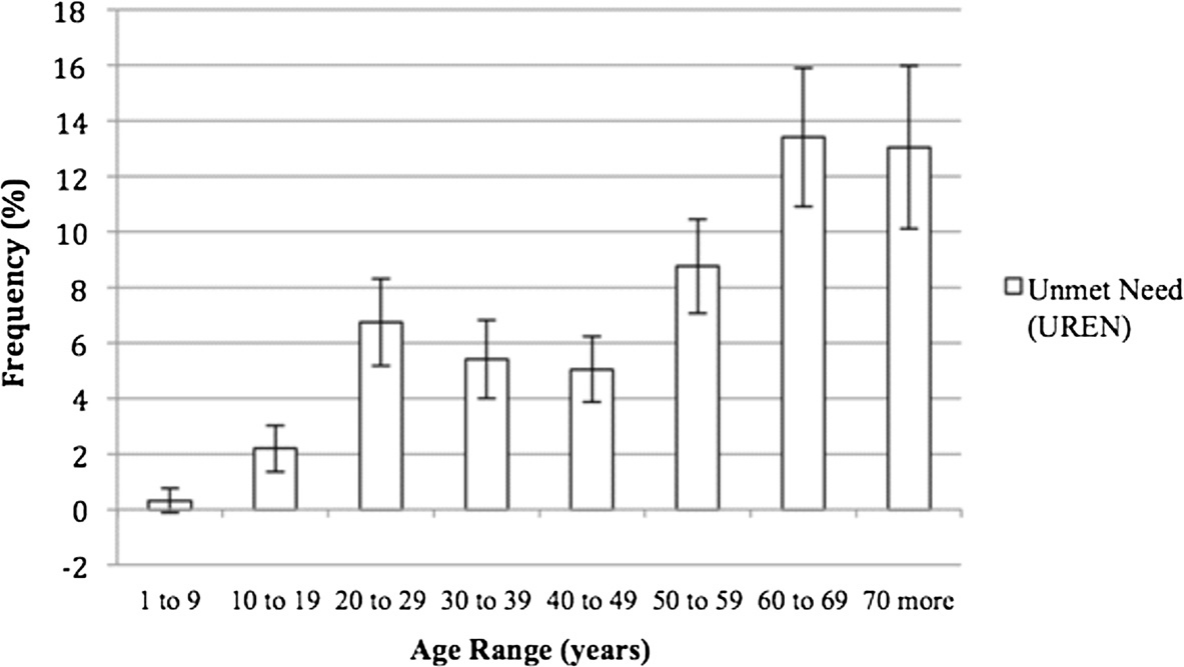
Prevalence of Unmet Need according to age in west region of state of São Paulo, Brazil – 2004/2005.

Amblyopia (BCVA > 0.15 logMAR) was present in at least one eye from 3% of children in the first decade of life. Considering the severe amblyopia (BCVA > 0.5 logMAR) 1,15% of eyes in the same age had this condition.Correction of RE provided greater benefit to individuals over 50 years and under 70 years of age (Figure 2).With respect to BCVA > 0.15 logMAR, 4.7% of the individuals were in the 1st age group, and there was a progressive increase in the following groups, reaching more than 63% after 70 years. BCVA > 1.3 logMAR represented a smaller percentage of persons compared to the other segments; 38.1% of individuals over 70 years of age maintained low vision or blindness (Figure 3).The prevalence of individuals with low vision and blindness who benefited from spectacles and who were removed from the category of extreme VI were also studied by measuring the difference between the frequencies of UCVA and BCVA to low vision (0.5 < AV ≤ 1.3 logMAR) and blindness (>1.3 logMAR). According to these criteria, prevalence of 4.6% of optically reversible low vision and 1.8% of blindness reversible by optical correction were found. Regarding age groups, these proportions were higher for subjects with low vision, especially after the age of 50, with nearly 12.5% having low vision that was reversible. For blindness, this difference was less significant, although there was a 4.9% decrease in the category with optical correction, as observed in individuals older than 70 years (Figure 4).The prevalence of UREN, represented by persons being removed from the category of VI with optical correction, was 6.5% (95% CI: 6.0-7.1) (Figure 5). There is a clear frequency for individuals in their 7th decade with 13.4% (95% CI: 10.9-15.9) and 8th decade with 13.0% (95% CI: 10.1-16.0), which reveals a greater benefit from refractive correction for these segments. Between 20 years and 29 years, this difference was 6.7% (95% CI: 5.2-8.3) (Figure 5).

UREN was evaluated according to the type of RE. The frequency of UREN for myopia, hyperopia and astigmatism of lower grades and for anisometropia remained between 7.8% and 13.2%. In patients with high myopia, 35.8% (95% CI: 30.5 - 41.0) were removed from the category of VI with optical correction, and for individuals with high hyperopia, the frequency of UREN was 34.5% (95% CI: 29.0 - 39.1).

### Univariate and multivariate analyses

The variables were included in logistic regression models using univariate analyses to establish the likelihood of association with the improvement of VA with RE correction. Considering URE and UREN as dependent variables, with the exception of self-reported skin color, all other related determinants were considered likely predictors (p < 0.001).The logistic regression models for URE and UREN, according to the association variables, are presented in Table 2.

**Table 2:** Results of multivariate analysis through logistic regression models for enhancement criteria of URE and UREN

	**URE OR (IC 95%)**	***P value***	**UREN OR (IC 95%)**	***P value***
**Gender**				
** Man**	0.71 (0.61 – 0.83)	< 0.001	0.67 (0.54 – 0.83)	< 0.001
** Woman**	1		1	
**Age**				
** 1 to 9**	0.14 (0.08 – 0.24)	< 0.001	0.02 (0.005 – 0.8)	< 0.001
** 10 to 19**	0.23 (0.16 – 0.34)	< 0.001	0.15 (0.09 – 0.24)	< 0.001
** 20 to 29**	0.44 (0.31 – 0.61)	0.03	0.4 (0.27 – 0.61)	0.16
** 30 to 39**	0.42 (0.30 – 0.58)	0.009	0.34 (0.22 – 0.51)	0.83
** 40 to 49**	0.61 (0.46 – 0.82)	0.21	0.40 (0.27 – 0.59)	0.15
** 50 to 59**	1.21 (0.92 – 1.59)	< 0.001	0.71 (0.50 – 1.00)	< 0.001
** 60 to 69**	1.77 (1.34 – 2.34)	<0.001	1.06 (0.75 – 1.48)	< 0.001
** 70 or +**	1		1	
**Background**				
** No background**	1.02 (0.87 – 1.20)	0.77	0.88 (0.71 – 1.10)	0.27
** Background**	1		1	
**Astigmatism axis**				
** Vertical**	1		1	
** Horizontal**	1.01 (0.84 – 1.22)	0.34	0.95 (0.74 – 1.21)	0.48
**Myopia**				
** Yes**	1		1	
** No**	0.19 (0.15 – 0.24)	< 0.001	0.1 (0.07 – 0.14)	< 0.001
**Hyperopia**				
** Yes**	1		1	
** No**	0.34 (0.28 – 0.42)	< 0.001	0.24 (0.17 – 0.34)	< 0.001
**Astigmatism**				
** Yes**	1		1	
** No**	0.6 (0.51 – 0.71)	< 0.001	0.83 (0.66 – 1.06)	0.13
**Anisometropia**				
** Yes**	1		1	
** No**	1.04 (0.85 – 1.26)	0.67	0.56 (0.44 – 0.70)	< 0.001

Following the criteria of URE or UREN, we found a significant association of prevalence with sex (p < 0.001), revealing a decrease of approximately 33% for men over women (URE:OR 0.7; 95% CI: 0.6-0.8; and UREN:OR 0.7, 95% CI: 0.5-0.8).

An association with age was also found. In individuals <50 years of age, the chance of URE was significantly lower than in those >60 years (p < 0.001), reaching nearly 1/10 individuals in the 1st age group (OR 0.1; 95% CI: 0.1-0.2) and 1/4 in the 2nd decade of life (OR 0.2; 95% CI: 0.2-0.3) compared to individuals >70 years of age. In individuals between the ages of 60 and 69 years, there was a higher frequency of persons with URE, and the frequency of BCVA for normal VA was approximately 80% more than for the last age group (OR 1.8; 95% CI: 1.3 - 2,3). Regarding UREN, significance was found only in the 1st and 2nd decades, when the chance of having UREN was much lower compared to individuals >70 years of age (OR 0.0; 95% CI: 0.005-0.8; and OR 0.1; 95% CI: 0.1-0.2, respectively).

For patients with RE, a significant association with SE (p < 0.001) was observed. When myopia was absent, there was an approximate 1/5 chance of URE and a 1/10 chance of UREN compared to the myopic carriers (OR 0.2; 95% CI: 0.2-0.2; OR, 0.1; 95% CI: 0.1-0.14, respectively). In the absence of hyperopia, the chance of URE and UREN was approximately 1/3 and 1/4 lower, respectively, compared to those for hyperopia carriers (OR 0.3; 95% CI: 0.3-0.4; OR 0.2; 95% CI: 0.2-0.3).

However, astigmatism demonstrated a significant association only with URE. When astigmatism was absent, the chance of URE was almost 1/2 compared to astigmatism (OR 0.6; 95% CI: 0.51-0.71). This significance was not observed for UREN, indicating that astigmatism alone was not sufficient to determine the optically correctable VI.

Optical correction exhibited a significant role in the reversal of VI for anisometropic carriers, with a lower chance of UREN for non-anisometropics, approximately two times lower than for those with anisometropia (OR 0.6; 95% CI: 0.4-0.7). There was no significant difference in the association between anisometropia and URE and there was no association between URE and UREN with regard to the systemic diseases and astigmatism cylindrical axes.

## Discussion

Initiatives to characterize the distribution profile of VI and identify reversible causes are fundamental for establishing preventive and therapeutic strategies to control the major causes of blindness.

An analysis of demographic data of the cities in the study stratified by age and gender shows that there was a predominance of women in almost all age groups and a flattening of men in the 3rd and 4rth age groups, most likely because the male is more likely to work in these age groups, reflecting a bias in sampling.

Approximately 70% of the participants had normal VA, requiring no refraction adjustment. Improving the VA to normal in 13.8% of the participants after BCVA corresponds to the prevalence of URE cases in this population that is a randomized sample of a Brazilian region.

Since there are many concepts in this topic, Table 3 presents the primary features regarding the definitions of VI, the different criteria used for classification of URE and UREN and their frequencies, thus establishing a parallel comparison with our and other studies worldwide [9,11,12,15-20]. Definitions represent a problem to compare studies. The prevalence of URE in Australia was 10.2% [11]. However, authors adopted 6/9 (0.18 logMAR) as the limit of normal VA, in contrast to the 0.15 logMAR adopted in our study. Another difference was the age groups. The Australian study included only individuals aged over 49 years and we considered all age groups to determine URE and considered vision improvement of two lines of the Snellen vision chart in the URE group, independent of the final level of VA [11]. In the current study, only the change of grade adopted was used as a criterion, which may influence the comparative analysis.

**Table 3:** Comparative analysis for results and criteria of URE, UREN and visual impairment between our and others populational surveys

**SURVEY (%)**	**URE**	**Criteria**	**UREN**	**Criteria**	**Low vision**	**Criteria**	**Blind**	**Criteria**
*Ferraz et al., 2014 São Paulo State, Brazil	13.8	Dif BCVA ≥ 0.15 - NCVA ≥ 0.15	6.5	Dif BCVA > 0.5 - NCVA > 0.5)	9.8	1.3 ≤ VA < 0.5	4.1	VA < 1.3
Ramke et al., 2012 [19] Timor-Leste, Afrique	3.7	NCVA < 6/18 ≥ 6/18 with pinhole	9.6	VA < 20/40 Enhancement 2 lines	-	-	-	-
Brian et al., 2011 [18] Figi, Japan	10.3	Presenting corrected vision ≥ 6/18	4.8					
Uribe et al., 2011 [9] Tucson/Nogales, USA	22.57	Enhancement 2 lines						
Barnes et al., 2011 [21] Ta’u Island, Samoa, USA	-	-	-	-	10.5	6/60 ≤ VA < 6/18	4.8	VA < 6/60
Schellini et al., 2009 [12] Botucatu Eye Study, Brazil	-	-	5.5	Dif BCVA ≥ 20/60 -NCVA ≥ 20/60)	5.2	20/400 ≤ VA < 20/60	2.2	VA <20/400
Varma et al., 2008 [16] La Puente, California	15.1	Enhancement 2 lines	8.9	Dif BCVA ≥ 20/40 - NCVA ≥ 20/40)	-	-	-	-
Ntim-Amponsah, 2007 [15] Gana, Afrique	11.9	Enhancement 2 lines	-	-	-	-	-	-
Ramke et al., 2007 [17] Timor-Leste, Afrique	-	-	11.7	Dif BCVA ≥ 6/18 - NCVA ≥ 6/18)	-	6/60 ≤ VA < 6/18	-	VA < 6/60
Dandona et al., 2002 [20] Andhra Pradesh, Índia	-	-	4.49	Dif BCVA ≥ 6/12 - NCVA ≥ 6/12)	-	VA < 6/12	-	-
Thiagalingam et al., 2002 [11] Blue Montains, Australia	10.2	VA < 6/9 Enhancement 2 lines	-	-	-	-	-	-

Note: *Ferraz et al.,2014 correspond to the present study. VA: 6/120 Sn = 20/400 Sn = 1.3 logMAR; 6/60 Sn = 20/200 Sn = 1.0 logMAR; 6/18 Sn = 20/60 Sn = 0.5 logMAR; 20/40 Sn = 0.3 logMAR; 6/9 Sn = 20/30 Sn = 0.18 logMAR.

Considering another region of the state of Sao Paulo, Brazil and specific age groups, we assessed the presenting vision of older adults and noted that the prevalence of blindness was 1.5%, which decreased to 1.1% with BCVA. In school children, the prevalence of uncorrected VI was 4.8%, which decreased to 12.4% with refractive correction [22].

A review of published data on URE as a cause of blindness and visual impairment in adults aged ≥40 years in sub-Saharan Africa (SSA) showed that the proportion of moderate VI (PVA ≤ 6/60 and >6/18) due to URE ranged from 12.3% to 57.1%. Although URE is a leading cause of VI, URE does not represent a major cause of blindness in SSA [23].

A Chinese study that used criteria similar to ours showed a prevalence of 24.8% for undercorrected vision; the met need was 10.4%, the URE was 13.2%, and the prevalence of mild visual impairment was 12.9% with presenting vision and 5.3% after BCVA [24].

The frequency of URE in another study involving individuals of all ages was 22.6%, considering the criteria for improvement of vision to be two or more lines with the BCVA for individuals with VA of 20/25 to 20/200 [9].

Our study showed that URE was present in all age groups, with a considerable increase in prevalence over 50 years (24% for patients over the age of 50 years), which reflects a greater benefit from optical correction after this age. Nevertheless, the frequency of individuals who have maintained some degree of VI, even with BCVA, was also proportionally larger in the elderly. We believe that with increasing age, the need for optical devices becomes greater; in addition, the presence of eye diseases that result in poor vision also increases.

The data for subjects with UREN showed that approximately 13% of individuals >60 years of age presented UCVA > 0.5 logMAR, i.e., individuals with VI no longer had this condition after corrective lenses. However, when severe VI or blindness is present, the likelihood of recovering VA with spectacles is lower because of the existence of important chronic degenerative diseases, such as diabetic retinopathy, glaucoma, cataracts, macular age-related degeneration, vitreo-retinal changes, corneal irregularities, high RE and amblyopia, which may present with severe VI and may not show significant improvement in VA with optical correction.

There is a direct association between the prevalence of blindness and VI with age, especially at advanced ages, confirming the presence of other problems contrary to visual improvement through refraction in the elderly. It is particularly startling that in “developed countries”, between 7% and 34% of older people have VI that could be reversed by appropriate spectacles. There is a strong relationship between impaired vision in older people and reduced quality of life and increased risk of accidents, particularly falls [25].

Brazil is a country of great miscegenation, which complicates the analysis of the influence of race. In the present study, we considered self-reported skin color, confirming that the majority of the population living in the state of Sao Paulo is white. The causes of blindness in the USA differ according to race; 50% of whites who are blind have macular degeneration related to age; in blacks, more than 33% of the causes of blindness result from cataracts; and among Hispanics, glaucoma causes blindness in 28% of the population; however, the primary condition related to VI in the three groups is cataracts [26].

A population‒based, cross‒sectional survey with cluster random sampling was used to select 50 clusters of 30 people over the age of 40 years in India; the survey demonstrated that “met refractive error need” in the sample was 2.2% and that UREN was 11.7% [17].

The prevalence of UREN found in this study was 6.5%, representing the proportion of individuals who were removed from the condition of VI with optical correction. In Bangladesh, analyzing more than 11,000 people, the prevalence of UREN was 7.2%, when limiting the VI to 6/12 (0.3 logMAR); using a new limit of 6/18 (0.5 logMAR), the same authors found a prevalence of 4.1% for UREN [2]. This analysis is similar to that used in our study and therefore allows a better comparison, thus revealing the major VI attributed to the lack of optical correction in Brazil.

However, in the USA, two criteria for UREN are considered: the BCVA necessary to obtain a driver’s license is 20/40 (0.3 logMAR) or the improvement of two lines of sight, regardless of prior VA, and the prevalence of UREN ranged from 8.9% to 9.6%. In both cases, the prevalence was higher than that reported in the present study. The discrepancy may be associated with the different parameters accepted for the definition of UREN. Compared to the findings in India [2], the prevalence of cases of UREN was higher in the USA [16].

Applying the criterion of VI recommended by the WHO, the prevalence of UREN in Timor Leste reached 11.7% [17]; and at Mount Figi, with a setting similar to the present study, the URE was 10.3%, whereas the UREN was 4.8% [18]. The prevalence of UREN in Timor-Leste was almost twice that found in the present study. This difference can be attributed to socioeconomic conditions because there is much poverty in Timor-Leste. Another aspect that contributes to this difference is that the sample in Timor-Leste consisted of individuals aged > 40 years [17], which shows a higher UREN; this was also observed in the current study, in which the prevalence of UREN in persons >60 years was approximately 13%.

Many factors and different definitions used in other population studies limit the usefulness of comparing the prevalence findings for UREN. Thus, a careful analysis of the sampling characteristics, regional differences, and socio-economic characteristics of the country and the study population demographics should be done. Furthermore, the criteria used to define VI are not uniform; thus, comparative analyses of the results may not be informative.

Regarding the type of ametropia, UREN cases were more prevalent among high RE (SE ≤ −3.00D and SE ≥ 3.00D). It is natural to expect that for higher refraction values, visual limitation is more pronounced, and cases without properly adjusted spectacles are expected to be more frequent. Among subjects with low RE, the lower limitation is the lack of spectacles, which is better tolerated.

It is important to remember that URE and UREN indirectly reflect the quality of health care and access to it. Many of the individuals who were examined in our study had not prior access to screening for RE, and a screening may have improved their VA condition. This fact must be acknowledged to guide the development of eye health programs.

## Conclusion

The analysis of prevalence and logistic regression models shows that 13.8% of the study participants exhibited improved VA with spectacles and that the vision of 6.5% with blindness and low vision was improved by optical correction. This important benefit was related to age, and was observed more frequently in persons over 50 years and with high RE. The data of our study point up the importance of refractive correction on VI.

## Abbreviations

BCVA: Best corrected visual acuity; CF: Count fingers; CI: Confidence interval; HM: Hands movements; LogMAR: Logarithm of minimal angle resolution; LP: Light perception; NCVA: No corrected visual acuity; NLP: No light perception; RE: Refractive error; SE: Spherical equivalent; URE: Uncorrected refractive error; UREN: Unmet refractive error; VA: Visual acuity; VI: Visual impairment; WHO: World Health Organization.

## Competing interests

All the authors declare that they have no competing interests.

## Authors’ contributions

FHF performed the acquisition, data analysis and writing. JEC and PO contributed to the analysis data. SAS was the responsible for the study design and revised the manuscript. All authors read and approved the final manuscript.

## Pre-publication history

The pre-publication history for this paper can be accessed here:

http://www.biomedcentral.com/1471-2415/14/84/prepub
